# Inflammatory Response Modulation by Vitamin C in an MPTP Mouse Model of Parkinson’s Disease

**DOI:** 10.3390/biology10111155

**Published:** 2021-11-09

**Authors:** Francesco De Nuccio, Antonia Cianciulli, Chiara Porro, Marianna Kashyrina, Melania Ruggiero, Rosa Calvello, Alessandro Miraglia, Giuseppe Nicolardi, Dario Domenico Lofrumento, Maria Antonietta Panaro

**Affiliations:** 1 Department of Biological and Environmental Sciences and Technologies, Section of Human Anatomy, University of Salento, I-73100 Lecce, Italy; francesco.denuccio@unisalento.it (F.D.N.); marianna.kashyrina@unisalento.it (M.K.); alessandro.miraglia@unisalento.it (A.M.); Giuseppe.nicolardi@unisalento.it (G.N.); 2 Department of Biosciences, Biotechnologies and Biopharmaceutics, University of Bari, I-70125 Bari, Italy; antonia.cianciulli@uniba.it (A.C.); melania.ruggiero@uniba.it (M.R.); rosa.calvello@uniba.it (R.C.); mariaantonietta.panaro@uniba.it (M.A.P.); 3 Department of Clinical and Experimental Medicine, University of Foggia, I-71100 Foggia, Italy; chiara.porro@unifg.it

**Keywords:** vitamin C, inflammasome, Parkinson’s disease, neuroinflammation, neuroprotection

## Abstract

**Simple Summary:**

Vitamin C (Vit C), also called ascorbic acid, is a nutrient present in many foods, particularly citrus fruits and green vegetables. Inadequate dietary Vit C intake causes hypovitaminosis resulting in the risk of developing clinical scurvy, potentially fatal if untreated. Vit C represents one of the safest and most essential nutrients, with antioxidant and anti-inflammatory properties that protect living organisms against oxidative stress; due to this propriety, it is studied for applications in the prevention and management of different pathologies, including neurodegenerative disease. Persistent neuroinflammation is detrimental for the brain and may lead to pathogenesis and progression of neurodegenerative diseases like Parkinson’s disease (PD) and Alzheimer’s disease. The role of Vit C in the central nervous system is still debated.This study, utilizing a PD mouse model, has demonstrated that Vit C reduces neuroinflammation by the modulation of microglial responses and astrocyte activation, reducing dopaminergic neuronal cell loss involved in PD insurgence.Furthermore, mouse gait and spontaneous locomotor activity were partially ameliorated. In summary, we have demonstrated that the use of Vit C has neuroprotective effects in the brain, alleviating the inflammatory cascade and reducing the progression of PD.

**Abstract:**

Vitamin C (Vit C) is anutrient present in many foods, particularly citrus fruits, green vegetables, tomatoes, and potatoes. Vit C is studied for its applications in the prevention and management of different pathologies, including neurodegenerative diseases. Neuroinflammation is a defense mechanism activated by a stimulus or an insult that is aimed at the preservation of the brain by promoting tissue repair and removing cellular debris; however, persistent inflammatory responses are detrimental and may lead to the pathogenesis and progression of neurodegenerative diseases like Parkinson’s disease (PD) and Alzheimer’s disease. PD is one of the most common chronic progressive neurodegenerative disorders, and oxidative stress is one of the most important factors involved in its pathogenesis and progression.Due to this, research on antioxidant and anti-inflammatory compounds is an important target for counteracting neurodegenerative diseases, including PD. In the central nervous system, the presence of Vit C in the brain is higher than in other body districts, but why and how this occurs is still unknown. In this research, Vit C, with its anti-inflammatory and anti-oxidative properties, is studied to better understand its contribution to brain protection; in particular, we have investigated the neuroprotective effects of Vit C in the 1-methyl-4-phenyl-1,2,3,6-tetrahydropyridine (MPTP)-induced animal model of PD and its role in the modulation of neuroinflammation. First, we observed that Vit C significantly decreased the MPTP-induced loss of tyrosine hydroxylase (TH)-positive dopaminergic neuronal cells in the substantia nigra, as well as microglial cell activation and astrogliosis. Furthermore, gait and spontaneous locomotor activity, evaluated by an automated treadmill and the Open Field test, respectively, were partially ameliorated by Vit C treatment in MPTP-intoxicated animals. In relation to neuroinflammation, results show that Vit C reduced the protein and mRNA expression of inflammatory cytokines such as IL-6, TLR4, TNF-α, iNOS, and CD40, while anti-inflammatory proteins such as IL-10, CD163, TGF-β, and IL-4 increased. Interestingly, we show for the first time that Vit C reduces neuroinflammation by modulating microglial polarization and astrocyte activation. Moreover, Vit C was able to reduce NLRP3 activation, which is linked to the pathogenesis of many inflammatory diseases, including neuroinflammatory disorders. In conclusion, our study provides evidence that Vit C may represent a new promising dietary supplement for the prevention and alleviation of the inflammatory cascade of PD, thus contributing to neuroprotection.

## 1. Introduction

Vitamin C (Vit C), also called ascorbic acid, is a lactone with six carbon atoms and is water-soluble with a molar mass of 176 Da. It represents one of the safest and most essential nutrients, with antioxidant properties that protect living organisms against oxidative stress [1].

Moreover, Vit C is a cofactor in many biochemical synthetic reactions, such as the synthesis of hormones and neuropeptides and in non-heme iron absorption.VitC normally circulates in the blood at a concentration of 40 –60μmol/L. Inadequate dietary intake determines hypovitaminosis C (established as a plasma concentration ≤ 23 μmol/L), resulting in the risk of developing clinical scurvy, which is potentially fatal if untreated. However, as little as 10 mg/day of Vit C is enough to prevent overt scurvy [2]. The majority of vertebrates and invertebrates can produce Vit C from glucose in the liver; however, anthropomorphic primates, guinea pigs, bats, and some bird species lost this ability due to mutations in the L-gulono-γ-lactone oxidase (Gulo) gene, which encodes a crucialfor Vit C biosynthesis [3]. Vit C uptake is guaranteed by specific transporter systems and actively guided by a sodium gradient. All cells express members of the transporter family SLC23, the sodium-dependent Vitamin C transporters 1 and 2 (SVCT1 and SVCT2) [4,5].

Being of low molecular weight, Vit C is freely filtered by the kidney but reabsorbed in the proximal renal tubule by SVCT1. Vit C is actively transported into all cells of the body (except erythrocytes) by the SVCT2, which is closely related to theSVCT1 in structure and function [6]. After absorption in the intestinal tract by the SVCT1, Vit C passes across the extracellular and intracellular compartments of the organism by the SVCT2. SVCT2 is closely related to SVCT1 in structure and function [6], exerting various beneficial effects, including protection against immune system deficiencies, cardiovascular disease, cartilage and bone maintenance, prenatal health problems, eye disease, and wound healing [7].

It is not entirely elucidated how ascorbic acid enters the central nervous system(CNS) [8]. In the brain, SVCT2 is present exclusively in neurons [5], hypothalamic glial cells [9], and epithelial cells from the choroid plexus [5].

In the CNS, the role played by Vit C is only partially established. In this regard, Vit C concentrations in the CSF appear higher (200–400 mM) in comparison to those observed in the nervous parenchyma and plasma (30–60 nM).

The brain uses 25% of the total body glucose [10]. Due to elevated activity and high oxidative metabolism, the brain needs antioxidants to be protected from oxidative stress [11].In fact, redox imbalance and oxidative stress are detected in neurodegenerative disorders such as Parkinson’s disease (PD), Alzheimer’s disease (AD), Huntington’s disease, Amyotrophic lateral sclerosis, among others [12]. In this regard, Vit C in the brain and CSF has been reported for its antioxidant and neuroprotective effects to oppose glutamate excitotoxicity [13].

Although Vit C has attracted particular attention due to its anti-inflammatory, immunomodulating, and antioxidantproperties [14], its effect on neuroinflammation is still unclear.

Neuroinflammation involvement in pathogenesis and progression of different neurodegenerative diseases, including AD, PD, HIV-associated dementia, and multiple sclerosis, has been established. In particular, several studies have suggested that free radicals, pro-inflammatory cytokines, and oxidative stress have a significantrole in PD pathogenesis [15,16].

PD is among the most common neurodegenerative disorders, in which a progressive loss of dopaminergic neurons in the substantia nigra parscompacta (SNpc)is seen, leading to movement disorders that progress over time. The etiology of the pathology is still unknown, but the neurodegenerative processes engage an array of different elements that influence each other. PD involves genetic, environmental, and toxicological factors, and both oxidative damage and mitochondrial dysfunction are involved in the cascade of events leading to the degeneration of dopaminergic neurons [17].

In accordance with our previous studies, intraperitoneal injections of the neurotoxin 1-methyl-4-phenyl-1,2,3,6-tetrahydropyridine (MPTP) can induce a PD disease picture in mice [18].

In the present research, we investigated the potential neuroprotective effects of Vit Cin a PD mouse model induced by MPTP with an assessment of mice locomotor activity, astrocytes, and microglia activation together with the characterization of dopaminergic neuronal degeneration in SNpc. The anti-inflammatory properties of Vit C have also been evaluated by monitoring the release of the mediators and the expression of markers involved in the inflammatory response.

In relation to neuroinflammation, results show that Vit C reduced the protein and mRNA expression of inflammatory cytokines such as IL-6, TLR4, TNF-α, iNOS, and CD40, while anti-inflammatory proteins such as IL-10, CD163, TGF-β, and IL-4 increased. 

Here we show for the first time that Vit C modulates neuroinflammation by regulating microglial polarization and astrocyte activation. Moreover, Vit C was able to reduce NLRP3 activation, which is linked to the pathogenesis of many inflammatory diseases, including neuroinflammatory disorders.

## 2. Materials and Methods

### 2.1. Animals and Treatment Protocols

In this experimental procedure, forty-eight male mice of the 129SV strain weighing between 22–26 g (Envigo, Udine, Italy) were used. Half of the mice were treated with vitamin C (15 mg/Kg; Sigma-Aldrich, Milan, Italy) daily for 10 days (intragastric gavage), while the rest of the mice received vehicle (water) only. The 15 mg/Kg dose of vitamin C is equivalent to 1 g for an adult human, a dosage that is within the range of the amount found in commercially available vitamin C supplements (500–1000 mg tabs), well below the upper limit considered safe for an adult (2 g/day). On the third day, 12 mice from both groups were treated with 4 doses of MPTP (20 mg/Kg) at two-hour intervals by intraperitoneal injection. Control mice received sterile saline solution. After 7 days, the mice were sacrificed [19]. Experimental procedures followed the protocols approved by the Institutional Animal Committee and in accordance with the European Union (EU) Directive 2010/63/EU for animal experiments.

### 2.2. Immunohistochemistry

An indirect biotin-avidin method was utilized for immunohistochemical procedures [20]. Sections of 10 μm, obtained by rotative microtome, were incubated with a 1:1000 solution of mouse primary monoclonal antibody (MoAb) anti-GFAP (Merck Millipore, Burlington, MA, USA), a 1:1000 solution of rabbit policlonal (p)Ab anti-Iba-1 (Fujifilm Wako Chemicals, Osaka, Japan), or a 1:1500 solution of anti-TH mouse MoAb (BioLegend, Amsterdam, The Netherlands) overnight. The following day, incubations were performed with biotinylated secondary antibody and then with extravidin-peroxidase, which binds to biotin. Subsequently, using the chromogenic substrate 3,3′-diaminobenzidine (DAB), a brown-colored oxidation product was visualized in correspondence of immunocomplexes. Images were acquired with a DS-5M digital camera assembled on a Nikon Eclipse E800 microscope (Nikon Instruments S.p.A, CampiBisenzio, FI, Italy).

### 2.3. Open Field Test

The Open Field test was utilized for the evaluation of locomotor activity and exploration habits in mouse and rat models of CNS disorders.

For the experimental procedures, a 20 × 20 cm box was used (arena). Mice were placed in the center and were allowed to explore the arena for 5 min;the movements were recorded by a camera andanalyzed by an automated tracking system (Panlab SMART, Harvard Apparatus, Holliston, MA, USA). The following parameters were analyzed, total distance traveled, distance traveled in the central area of the arena (central zone), time spent in the central zone, and number of entries in central zone. Between trials, the arena was wiped with 70% alcohol and carefully dried.Tests were conducted for six days after MPTP treatment between 9:00 a.m. and 12:00 p.m.

### 2.4. Automated Treadmill Gait Test

Treadmill gait evaluation was carried out with the DigiGait imaging apparatus (Mouse Specifics Inc., Framingham, MA, USA) (see [21,22] for details).

Mice were positioned on a motorized treadmill that was set at a speed of 20cm/sec, as suggested by the manufacturer. The videos (30 s duration at 80 FPS), recorded from a camera located under the treadmill, were analyzed by the software (a time frame of 5 s), which automatically identifies the paw footprints and generates digitized “fingerprints of the paw” that are subsequently processed to calculate the parameters relating to the animal’s gait. The following parameters were taken into consideration:swing time, stance time, stance/swing ratio, stride duration, stride frequency, and paw angle.

### 2.5. Immunoblotting

Striatum and SNpcwere collected following the procedure described by Jackson-Lewis and Przedborski [19] and homogenized in a lysis buffer containing 50 mM Tris pH 8, 1% Triton-X, 0.2% sodium dodecyl sulfate (SDS), 1.5 M NaCl supplemented with protease inhibitors Aprotinin (4 U/mL), Leupeptin (2µM), and PMSF (100 µM). Multiple cycles of freezing and thawing were completed to improve lysis, and the subsequent lysate was centrifuged at 13,000× *g* for 20 min at 4 °C. The protein quantification was performed with Bradford protein assay by measuring the absorbance of samples at 595 nm and interpolating the optical density with a calibration line. A quantity of 25 μg of proteins from each sample was uploaded on 4–12% precast polyacrylamide gels and fractionated in relation to the size by applying a voltage of 200 V.

The bands were electrophoretically transferred on nitrocellulose membranes, then incubated with anti-GFAP mouse MoAb (Millipore), anti-TH mouse MoAb (Millipore), anti-Iba-1 mouse MoAb (Wako, Neuss, Germany), anti-IL-10rabbit pAb, anti-IL-6rabbit pAb, anti-TLR4mouse MoAb, anti-TNF-α mouse MoAb, anti-NOS2 (iNOS)mouse MoAb, anti-NOS2 (iNOS)mouse MoAb, anti-CD40mouse MoAb, anti-TGF-βmouse MoAb, anti-CD163mouse MoAb, anti-IL-4mouse MoAb, anti-NLPR3mouse MoAb, anti-pIKb mouse MoAb (all from Santa Cruz Biotechnology, Inc., Milan, Italy). β-actin was used as an internal control. The detection of proteins was completed via the chemiluminescence method (BioRad, Milan, Italy), exploiting the binding of primary antibodies HRP-conjugated secondary antibodies (Santa Cruz Biotechnology, Inc., Milan, Italy). The protein bands were scanned, and the optical density was determined by using the Kodak 1Dimage analysis software (Kodak, Rochester, NY, USA). The normalization was done with β-actin, and the results, expressed as means ± SD, were provided as relative optical density.

### 2.6. Real-Time PCR

Tissues were treated with the TRIzol reagent (Invitrogen, Milan, Italy)according to the manufacturer’s protocol in order to isolate total RNA, which was subsequently reverse-transcribed into cDNA by using the SuperScript™ III Reverse Transcriptase (Invitrogen, Milan, Italy).The cDNAs of TH, GFAP, Iba1, IL-6, TLR4, TNF-α, iNOS, CD40, IL-10, CD163, TGF-β, and IL-4 were amplified with a 7300 Real-Time PCR System (Life Technologies) together with the reference cDNA of GAPDH by using probes marked with a 5′ fluorescent dye (6-FAM) and a 3′ quencher dye (NFQ). The fluorescence emitted by samples was followed in real-time with an ABI PRISM 7300-sequence detection system (Applied Biosystems, Waltham, MA, USA), and the quantitative analysis was conducted comparing the threshold cycle (Ct) value of samples with that of the reference gene. The results were displayed in fold difference. Primer sequences used for the target amplifications are reported in Table 1.

### 2.7. Statistical Analysis

To determine the significance of data, a statistical analysis was carried out with Statgraphics Centurion software (Statgraphics Technologies Inc., The Plains, VA, USA). After checking the data analysis of variance (ANOVA) assumptions with the Shapiro-Wilk and Bartlett Test, the parametric ANOVA was performed, followed by the Dunnet post-hoc test. The statistical significance was demonstrated with a *p*-value < 0.05. 

## 3. Results

### 3.1. Effects of Vit C on TH Expression

**Figure 1:**
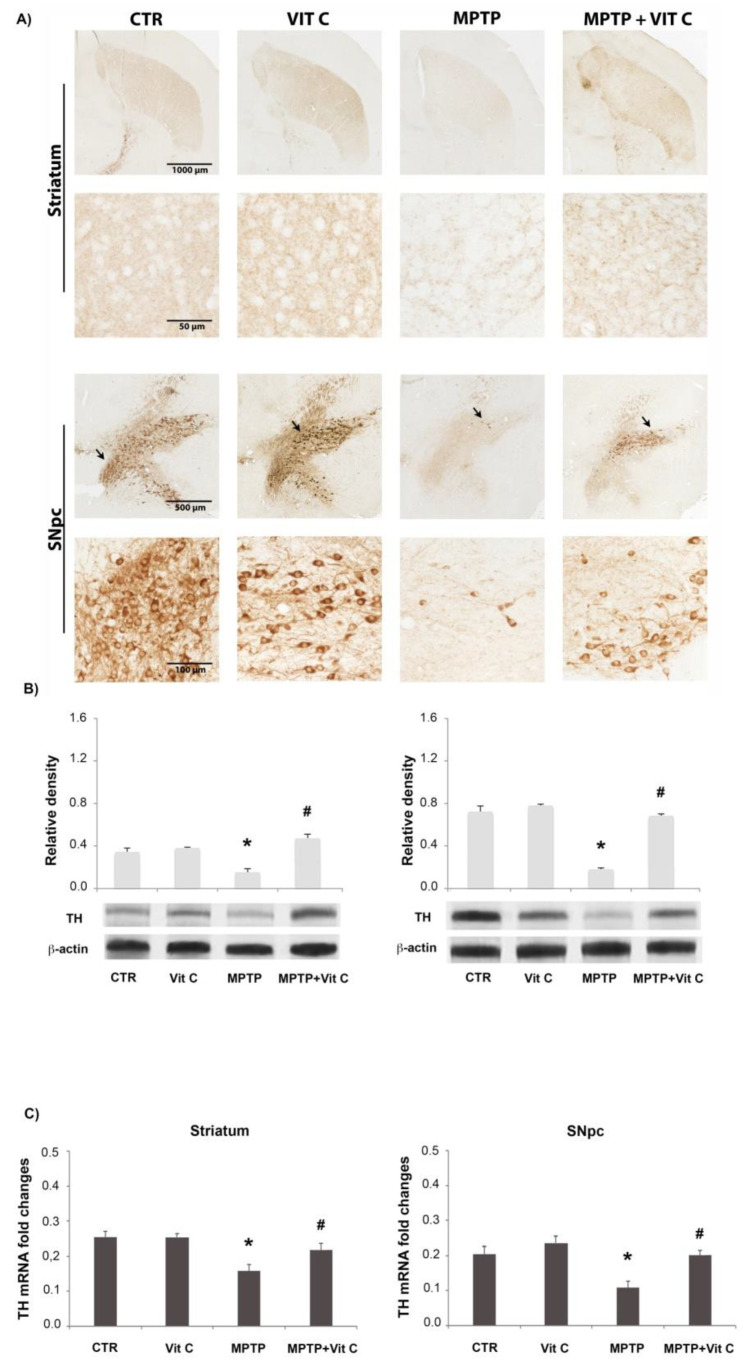
Tyrosine hydroxylase (TH) analysis. (**A**) TH immunoreactive catecholaminergic neurons of SNpcand their projections in the striatum (upper) and SNpc (lower) in controls (CTR), mice treated with Vitamin C (VIT C), MPTP, MPTP, and Vitamin C (MPTP+ VIT C). Scale bar: (striatum) upper 1000 μm (objective 4×), lower 50 μm (obj. 40×); (SNpc) upper 500 μm (obj. 4×), lower 100 μm (obj. 20×). In low magnification images, arrows point to TH immunoreactive cells shown in high magnification ones. (**B**) Western blotting detection and densitometric analysis of TH expression levels in controls (CTR), mice treated with Vitamin C (VIT C), MPTP, MPTP, and Vitamin C (MPTP+ VIT C) in striatum (**left**) and SNpc (**right**). Protein expression analysis values are expressed as arbitrary units after normalization against β-actin, using as a loading control (*n* = 5 in each group, 5 replicates). (**C**) Real-time PCR analysis of TH mRNA expression levels in controls (CTR), mice treated with Vitamin C (VIT C), MPTP, MPTP, and Vitamin C (MPTP+ VIT C) in striatum (**left**) and SNpc (**right**). Results are presented as mRNA fold changes relative to GAPDH, used as resident control. Data are presented as mean ± SD (* *p* < 0.05 vs. CTR; # *p* < 0.05 vs. MPTP). Supplementary Figure S1 shows the full western blots of Figure 1.

The expression of TH, the enzyme that catalyzes the rate-limiting step of dopamine(DA) biosynthesis, was assessed to evaluate the neuroprotective action of Vit C on SNpc DA neurons (Figure 1). MPTP administration induced a decrease in TH immunoreactivity (Figure 1A) in striatum and SNpc with respect to controls. In mice treated with MPTP that received Vit C, TH immunoreactivity was more intense compared to animals that received only MPTP, indicating that Vit C reduces dopaminergic neurons loss induced by MPTP. The TH immunoreactivity pattern in animals treated with Vit C was similar to controls (Figure 1A). TH protein expression analysis (Figure 1B) supported these observations because in the brains of animals treated with MPTP, TH loss was reduced significantly by Vit C administration. Similarly, TH transcript expression resulted in a significant increase in Vit C treated animals compared with animals treated with MPTP alone (Figure 1C).

### 3.2. Effects of Vit C on GFAP Expression

**Figure 2:**
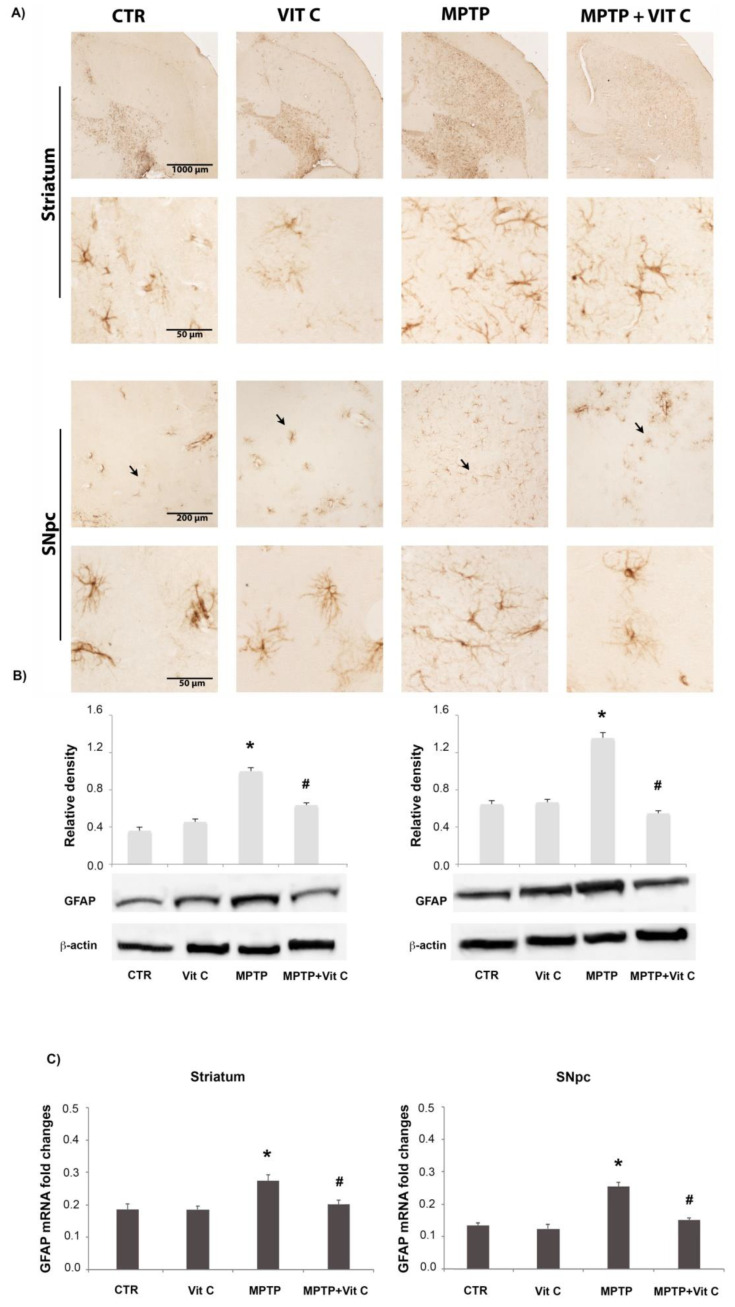
GFAP analysis. (**A**) GFAP immunoreactive astrocytes in the striatum (upper) and SNpc (lower) of controls (CTR), mice treated with Vitamin C (VIT C), MPTP, MPTP, and Vitamin C (MPTP+ VIT C). Scale bar: (striatum) upper 1000 μm (obj. 4×), lower 50 μm (obj. 40×); (SNpc) upper 200 μm (obj. 4×), lower 50 μm (obj. 40×). In low magnification images, arrows point to GFAP immunoreactive cells shown in high magnification ones. (**B**) Western blotting detection and densitometric analysis of GFAP expression levels in controls (CTR), mice treated with Vitamin C (VIT C), MPTP, MPTP, and Vitamin C (MPTP+ VIT C) in striatum (**left**) and SNpc (**right**). Protein expression analysis values are expressed as arbitrary units after normalization against β-actin, used as a loading control (*n* = 5 in each group, 5 replicates). (**C**) Real-time PCR analysis of GFAP mRNA expression levels in controls (CTR), mice treated with Vitamin C (VIT C), MPTP, MPTP, and Vitamin C (MPTP+ VIT C) in striatum (**left**) and SNpc (**right**). Results are presented as mRNA fold changes relative to GAPDH, used as resident control. Data are presented as mean ± SD (* *p* < 0.05 vs. CTR; # *p* < 0.05 vs. MPTP). Supplementary Figure S2 shows the full western blots of Figure 2.

GFAP expression was utilized as a marker of astroglialactivation (Figure 2). MPTP treatment induced a robust astrocyte activation, as demonstrated by the increase of GFAP immunoreactive cells as well as of staining intensity (Figure 2A); the administration of Vit C in MPTP-treated animals decreased GFAP immunoreactivity in striatum and SNpc. In western blot experiments (Figure 2B), the densitometric analysis of GFAP protein bands showed that MPTP treatment induced a significant increase of protein expression that was significantly reduced by Vit C administration. RT-PCR results showed a significant increase in mRNA expression following MPTP treatment (Figure 2C) in mice receiving Vit C administration.

### 3.3. Effects of Vit C on Microglia

**Figure 3:**
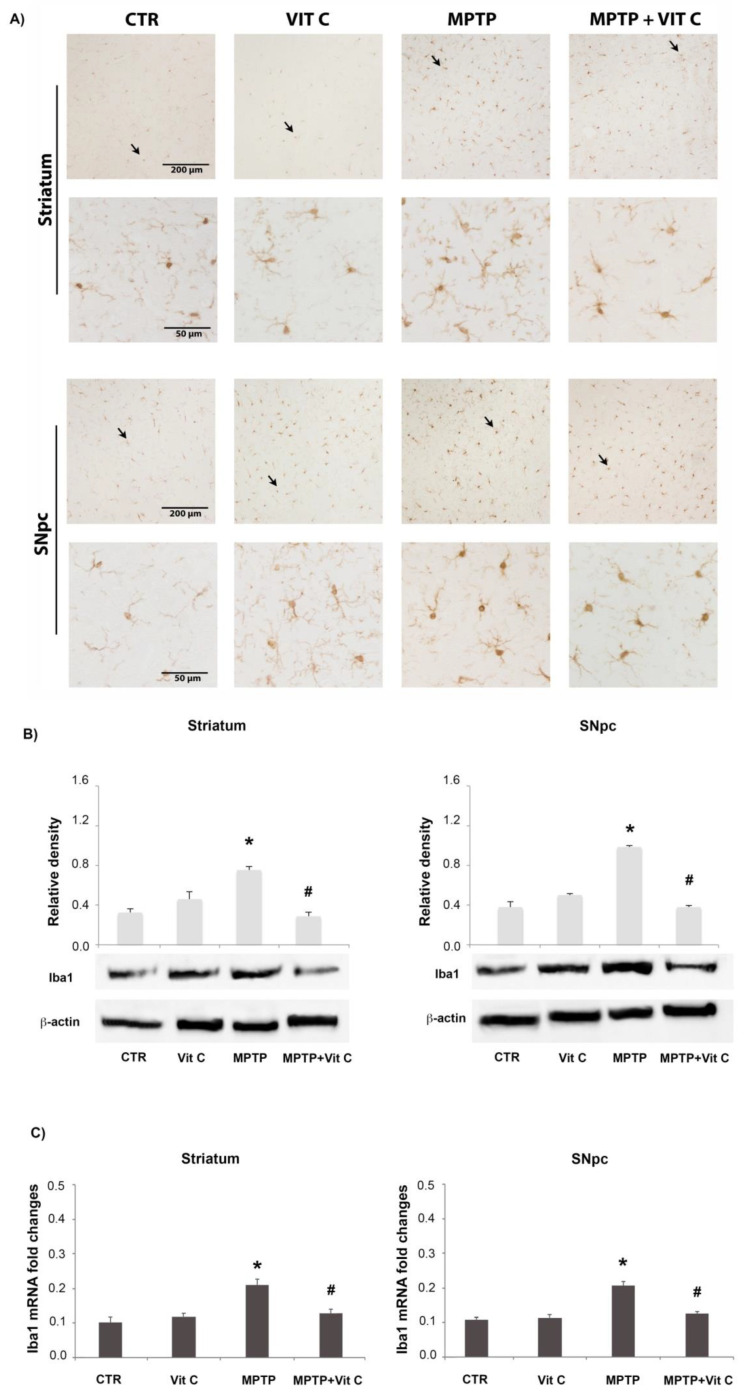
Iba1 analysis. (**A**) Iba1 immunoreactive microglial cells in striatum (upper) and SNpc (lower) of controls (CTR), mice treated with Vitamin C (VIT C), MPTP, MPTP, and Vitamin C (MPTP+ VIT C). Scale bar: (striatum) upper 200 μm (obj. 10×), lower 50 μm (obj. 40×); (SNpc)upper 200 μm (obj. 10×), lower 50 μm (obj. 40×). In low magnification images, arrows point to Iba1 immunoreactive cells shown in high magnification ones. (**B**) Western blotting detection and densitometric analysis of Iba1 expression levels in controls (CTR), mice treated with Vitamin C (VIT C), MPTP, MPTP, and Vitamin C (MPTP+ VIT C) in striatum (**left**) and SNpc (**right**). Protein expression analysis values are expressed as arbitrary units after normalization against β-actin, used as a loading control (*n* = 5 in each group, 5 replicates). (**C**) Real-time PCR analysis of Iba1 mRNA expression levels in controls (CTR), mice treated with Vitamin C (VIT C), MPTP, MPTP, and Vitamin C (MPTP+ VIT C) in striatum (**left**) and SNpc (**right**). Results are presented as mRNA fold changes relative to GAPDH, used as resident control. Data are presented as mean ± SD (* *p* < 0.05 vs. CTR; # *p* < 0.05 vs. MPTP). Supplementary Figure S3 shows the full western blots of Figure 3.

In order to evaluate the effects of Vit C treatment on microglia, Iba1 was used as a marker for these cells (Figure 3). In controls, few resting microglia were observed. Following MPTP injection, more Iba1-positive microglial cells were observed, and they showed a rounder shape (Figure 3A). On the other hand, in mice treated with MPTP and Vit C, the amount of activated microglia was reduced. Western blotting analysis showed that in Vit C-treated mice, there were comparable Iba1 levels to those in controls (Figure 3B). Iba1 protein expression resulted in significantly augmented MPTP-treated mice with respect to controls; interestingly, in MPTP mice treated with Vit C, Iba1 was significantly reduced with respect to animals that received onlyMPTP. Real-time PCR analysis confirmed these results; in fact, the mRNA levels were higher inMPTP-treated mice with respect to controls. Moreover, Vit C was able to reduce the expression of Iba1 mRNA levels in MPTP-treated animals compared with animals treatedwith MPTP alone (Figure 3C).

### 3.4. Exploratory Activity

**Figure 4:**
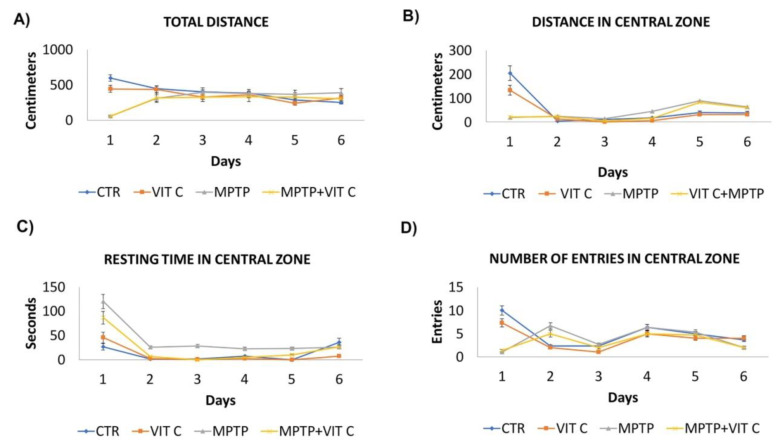
Locomotory and exploratory activity. The data show the total distance traveled (**A**), distance traveled in the central zone (**B**), time spent in central zone (**C**) and number of entries in central zone (**D**) in controls (CTR), mice treated with vitamin C (Vit C), MPTP, MPTP, and Vitamin C (MPTP+Vit C). All values are expressed as means ± SD (*n* = 5 per group, 5 replicates, * *p* < 0.05 MPTP+Vit C vs. Vit C, MPTP vs. CTR).

The Open Field test was performed to examine exploratory and gross locomotor activities. The total distance traveled, time spent in central zone, distance traveled in the central zone, and number of entries in the central zone were analyzed (Figure 4). As expected, the values of all parameters decreased from day 1 to day 6, because mice on the first day explored the new space, but in the following days, they quickly moved to the periphery and remained close to the walls. On the first day after MPTP treatment, both MPTP and MPTP+VitC-treated animals showed a significant reduction of exploratory activity with respect to controls and Vit C-only treated mice, as an effect of neurotoxic insult (MPTP+Vit C vs. Vit C, MPTP vs. CTR *p* < 0.05).Starting from day two, the locomotor behavior of MPTP and MPTP+Vit C treated mice improved (except for total distance), and in the next days, no significant differences among the groups were observed, except for the time spent in the central zone, whichwas significantly higher in MPTP-only treated animals compared to both controls (CTR, Vit C) and MPTP+Vit C (*p* < 0.05), suggesting that Vit C administration improved their ability to reach the peripheral zone.

### 3.5. Gait Dynamic Analysis

**Figure 5:**
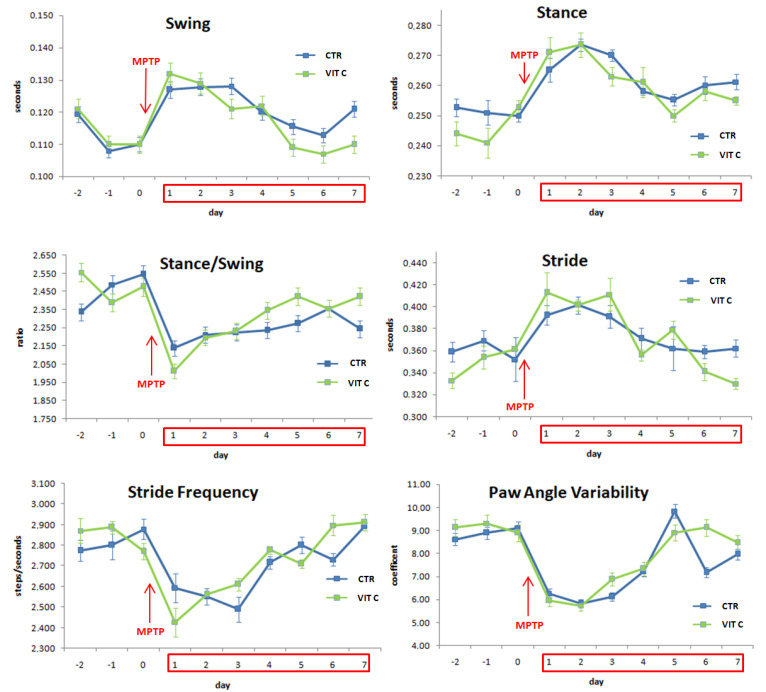
Treadmill-based measurement of gait properties. Plots showing swing time, stance time, stance/swing ratio, stride duration, stride frequency, and paw angle measured in mice treated with Vitamin C (Vit C) or untreated. On day 0, MPTP was administered to all mice. All values are expressed as means ± SD (*n* = 5 per group, 5 replicates, * *p* < 0.05 day 0 vs. day 1, # *p* < 0.05 Vit C vs. CTR).

Representative gait dynamic parameters of mice walking at a speed of 20 cm/s are reported in Figure 5. Gait recording started two days (day-2) before MPTP injection (day 0). On day 0,the recording was performed before the injection; recordings continued until animals were sacrificed (day 7 after MPTP treatment). Swing time, time the paw was not in contact with the belt, was significantly increased after MPTP-treatment, as well as stance time, the duration of time the paw is in contact with the belt (day 1 vs. day 0, *p* < 0.05). The stance/swing ratio decreased, suggesting that the swing time increased more than stance time. Stride was also affected, as demonstrated by the analysis of stride frequency and stride duration parameters: following MPTP administration, stride frequency was significantly reduced while its duration was augmented, as well as its length (day 1 vs. day 0, *p* < 0.05). The variability of the paw angle, i.e., the degree of the hind paw external rotation as a function of stride, was altered, too. Other gait parameters, such as brake, propel, stance width, and paw area, were not changed by MPTP intoxication (data not shown). Taken together, these results show that MPTP determined a change in mice motor behavior since stride length and duration were increased, while stride frequency was decreased; the latter can be attained by the increase of stance and swing duration. Vit C administration in mice determined some changes in gait parameters with respect to untreated mice several days after MPTP intoxication. In this context, swing and stance duration were significantly reduced from day 5, while stance/swing ratio increased from day 4 (Vit C vs. CTR *p* < 0.05). In Vit C-treated animals, the duration and frequency of stride, as well as paw angle variability, were significantly different from controls from day 5 (Vit C vs. CTR *p* < 0.05).

### 3.6. Effects of Vit C on the Expression of Pro- and Anti-Inflammatory Markers in MPTP-Treated Mice

**Figure 6:**
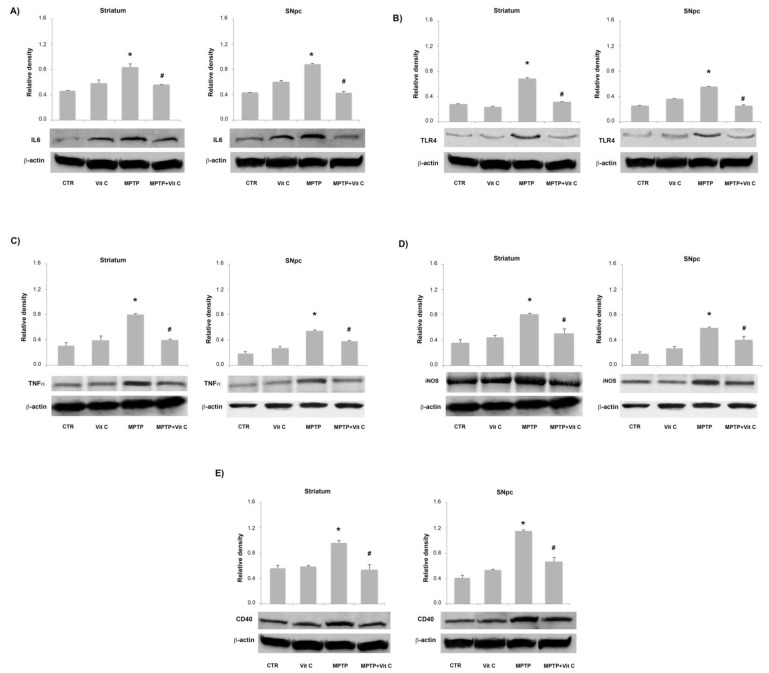
Effects of Vit C on the expression of pro-inflammatory markers in MPTP-treated mice. Western blotting detection and densitometric analysis of (**A**) IL-6, (**B**) TLR4, (**C**) TNF-α, (**D**) iNOS, and (**E**) CD40 expression levels in controls (CTR), mice treated with Vitamin C (VIT C), MPTP, MPTP, and Vitamin C (MPTP+ VIT C) in striatum (**left**) and SNpc (**right**). Protein expression analysis values are expressed as arbitrary units after normalization against β-actin, used as a loading control (*n* = 5 in each group, 5 replicates). Data are presented as mean ± SD (* *p* < 0.05 vs. CTR; # *p* < 0.05 vs. MPTP). Supplementary Figure S4 shows the full western blots of Figure 6.

**Figure 7:**
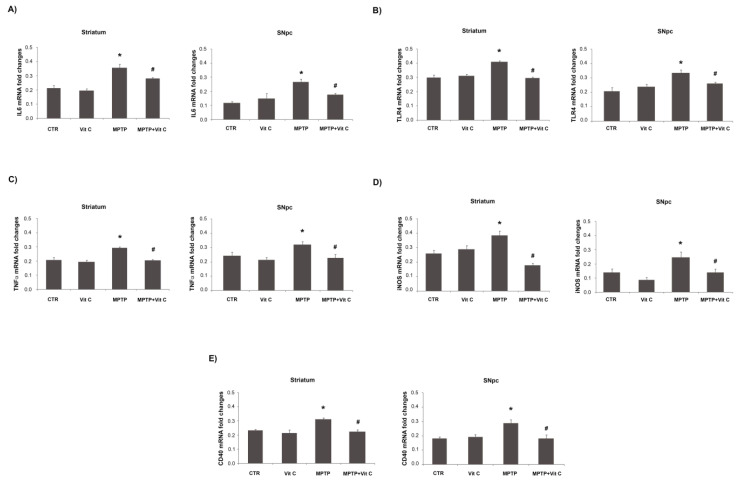
Effects of Vit C on the expression of pro-inflammatory markers in MPTP-treated mice. Real-time PCR analysis of (**A**) IL-6, (**B**) TLR4, (**C**) TNF-α, (**D**) iNOS, and (**E**) CD40 mRNA expression levels in controls (CTR), mice treated with Vitamin C (VIT C), MPTP, MPTP, and Vitamin C (MPTP+ VIT C) in striatum (**left**) and SNpc (**right**). Results are presented as mRNA fold changes relative to GAPDH, used as resident control. Data are presented as mean ± SD (* *p* < 0.05 vs. CTR; # *p*< 0.05 vs. MPTP).

Pro-inflammatory markers, in terms of IL-6, TLR4, TNF-α, iNOS, and CD40 expression, were detected by RT-PCR and immunoblotting analysis, at the level of both striatum and SNpc, in allanimals tested. Densitometric analysis of immunoblotting bands showedthat Vit C caused a significant reduction of protein expression levels both in striatum and SNpcin MPTP-treated animals with respect to MPTP-only treatedmice (Figure 6). These results were confirmed by quantitative PCR analysis, which revealed that in MPTP-treated animals, Vit C was able to significantly reduce mRNA pro-inflammatory markers in comparison to mice treated with MPTP alone (Figure 7).

**Figure 8:**
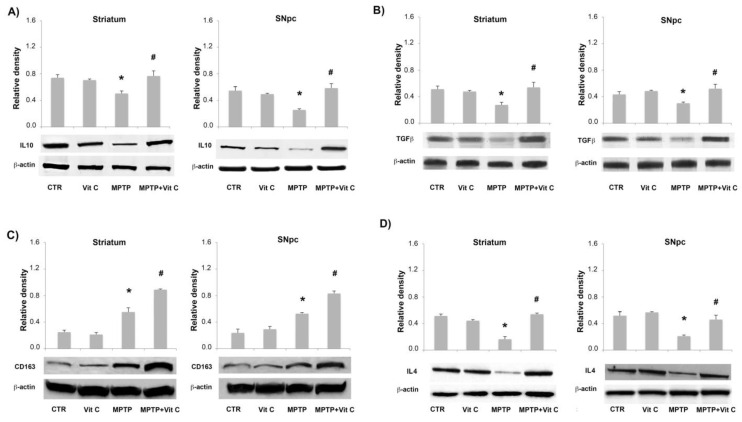
Effects of Vit C on the expression of anti-inflammatory markers in MPTP-treated mice. Western blotting detection and densitometric analysis of (**A**) IL-10, (**B**) TGF-β, (**C**) CD163, and (**D**) IL-4 expression levels in controls (CTR), mice treated with Vitamin C (VIT C), MPTP, MPTP, and Vitamin C (MPTP+ VIT C) in striatum (**left**) and SNpc (**right**). Protein expression analysis values are expressed as arbitrary units after normalization against β-actin, used as a loading control (*n* = 5 in each group, 5 replicates). Data are presented as mean ± SD (* *p* < 0.05 vs. CTR; # *p* < 0.05 vs. MPTP). Supplementary Figure S5 shows the full western blots of Figure 8.

**Figure 9:**
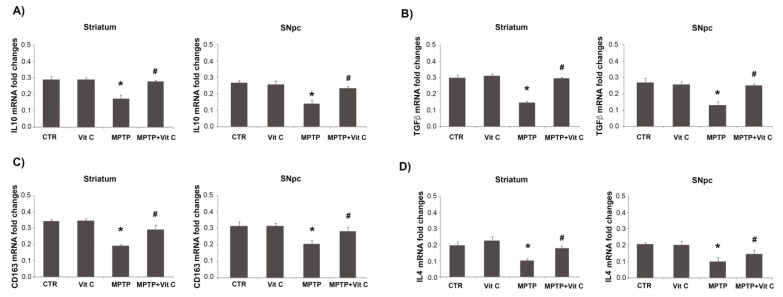
Effects of Vit C on the expression of anti-inflammatory markers in MPTP-treated mice. Real-time PCR analysis of (**A**) IL-10, (**B**) TGF-β, (**C**) CD163, and (**D**) IL-4 mRNA expression levels in controls (CTR), mice treated with Vitamin C (VIT C), MPTP, MPTP, and Vitamin C (MPTP+ VIT C) in striatum (**left**) and SNpc (**right**). Results are presented as mRNA fold changes relative to GAPDH used as resident control. Data are presented as mean ± SD (* *p* < 0.05 vs. CTR; # *p* < 0.05 vs. MPTP).

To test a possible anti-inflammatory regulation in the brains of mice treated with Vit C, we analyzed both proteins and mRNA expression of IL-10, CD163, TGF-β, and IL-4 in the striatum and SNpc. Figure 8 shows that the protein level expression of these markers was significantly up-regulated in Vit C-treated animals with respect to MPTP-treated ones. These results were confirmed by quantitative PCR analysis, as reported in Figure 9.

**Figure 10:**
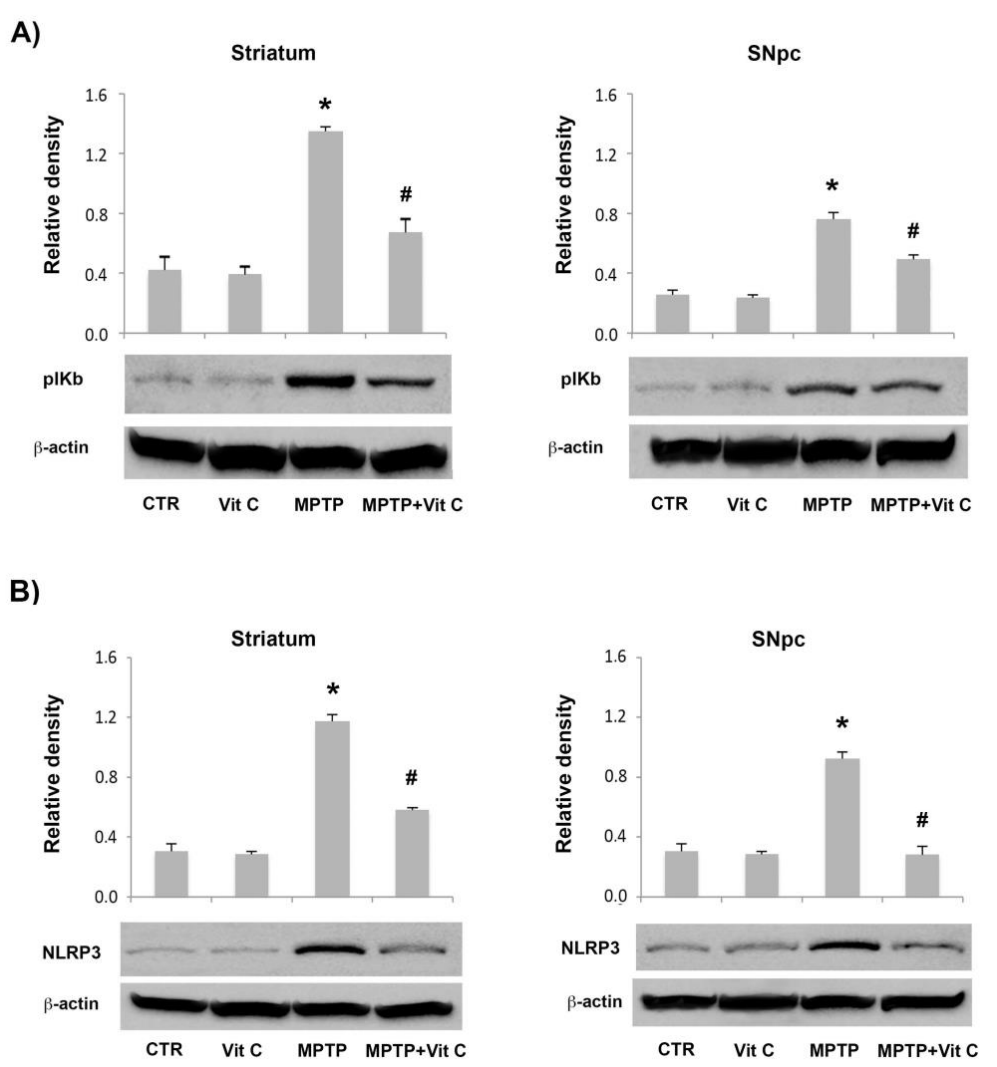
Western blotting detection and densitometric analysis of (**A**) pIKb and (**B**) NLRP3 expression levels in controls (CTR), mice treated with Vitamin C (VIT C), MPTP, MPTP, and Vitamin C (MPTP+ VIT C) in striatum (**left**) and SNpc (**right**). Protein expression analysis values are expressed as arbitrary units after normalization against β-actin, used as a loading control (*n* = 5 in each group, 5 replicates). Data are represented by mean ± SD (* *p* < 0.05 vs. CTR; # *p* < 0.05 vs. MPTP). Supplementary Figure S6 shows the full western blots of Figure 10.

Interestingly, our data show that NF-κB phosphorylation was strongly decreased by the addition of Vit C in MPTP-treated mice in comparison with the mice treated with MPTP alone, as reported in Figure 10A.

Finally, we also observed that Vit C was able to reduce the expression of NPLR3, a typical hallmark of inflammatory activation of microglia, in the MPTP mouse (Figure 10B). Taken together, these results demonstrate that Vit C is able to reducepro-inflammatory processes and up-regulate anti-inflammatory processes, thus playing a neuroprotective role in our PD animal model.

## 4. Discussion

In the CNS, the role of Vit C is still debated; to deepen our understanding of its contribution to neuroprotection, we have investigated its effects in a mouse PD model.

The results of this study show that Vit C exerts a protective role in MPTP-induced PD mice, as demonstrated by the attenuation of dopaminergic neuronal degeneration in SNpc and the reduction of striatal TH immunoreactive fiber loss. Gait disturbances and movement impairment were also partially reduced in Vit C-treated animals. In particular, the MPTP intoxication determined an increase of stride length and duration, while stride frequency was decreased, suggesting that the animals lengthened their stride. Previous works showed an increase of stride frequency following MPTP administration [21]; however, the authors set the treadmill speed at 34 m/s, whereas we chose 20 m/s on the basis of our own experiments, determining that at higher speed for MPTP-treated mice was difficult and caused the gait to become irregular. Vit C accelerated the recovery from neuronal damage since swing, and stance duration were significantly reduced from day 5, stride frequency was reduced from day 6, and stance/swing ratio increased on days 4, 6, and 7. We also evaluated spontaneous locomotor activity in an open field test by measuring the total distance traveled, the time spent in central zone, the distance traveled in the central zone, and number of entries in central zone. Following MPTP treatment, animals showed a significant reduction in exploratory activity with respect to controls. In the following days, as expected, the overall locomotor activity of intoxicated mice improved; in particular, Vit C administration improved their ability to reach the peripheral zone.

Together with these results, we observed a significant reduction of the inflammatory reactions and an inhibition of NLRP3 inflammasome activation. In fact, NLRP3 inflammasome activation observed in the brain of the MPTP-induced PD mice was significantly decreased in Vit C-treated mice, thus suggestingthat NLRP3-mediated neuro-inflammation is crucial to explain the neuroprotective effects of Vit C in our PD animal model.

PD is a neurodegenerative disorder of the extrapyramidal system characterized by motor symptoms such as akinesia, bradykinesia, tremor, rigidity, postural and gait impairment [23]. Progressive loss of midbrain dopaminergic neurons in the substantia nigra and axonal terminals in the striatum gives rise to characteristic motor disturbances [24]. Although the underlying mechanisms are still unknown, increasing studies report neuroinflammation as a crucial factor in the pathogenesis of PD, evidencing that inflammatory factors are closely related to the dopaminergic neuron loss in PD [25,26]. For this reason, targeting neuroinflammation may provide neuroprotection in PD.

The canonical signs of neuroinflammation are represented by elevated levels of pro-inflammatory mediators, including TNF-α, IL-1β, andIL-6 detected in the brains of PD patients [27]. Elevated expressions of IL-1βhave been described both in the brain and in the peripheral tissues of PD patients, aswell as in animal models [28]. In this context, IL-1β seems to be a key contributor to PD initiation and progression [29].

Several studies report that the activation of the nucleotide-binding oligomerization domain leucine-rich repeat and pyrin domain-containing protein 3 (NLRP3) inflammasome, one of the most intensively investigated inflammasomes, is detrimental in neurological diseases, including PD. NLRP3 inflammasome activation was detected both in the serum of PD patients as well as in the midbrains of PD model mice [30,31]. In AD-associated neuroinflammation, NLRP3 inflammasome was activated, as reported by Heneka et al. [32].

Both the NLRP3 inflammasome and TLR4 signaling are involved in tissue injury. The NLRP3 inflammasome and TLR4 signaling promote the inflammatory response leading to an increase in the release of proinflammatory cytokines, including IL-1β,IL-6, and TNF-α [33,34]. Thus, inhibition or decrease in the NLRP3/TLR4 signaling pathway is a promising potential target in several inflammatory-based chronic diseases [35].

Interestingly, data obtained in the present study evidenced that treatment with Vit C attenuated neuroinflammation responses in mice by reducing the expression of the axis TLR4/NF-κB/NLRP3/1/IL-1βas reported in the Section 3. In this regard, the activation of the NLRP3 inflammasome complexes is involved regulation of IL-1β; thus, the subsequent downregulation of this signal pathway leads to IL-1β release reduction.

In agreement with our results, previous reports demonstrated this anti-inflammatory effect of Vit C, although in different experimental models, by modulating the expression levels of the NLP3 inflammasome with subsequent IL-1β reduction, through the TLR4/MyD88/NF-κB signaling pathway [36,37,38].

It is well documented that reactive astrocytes and activated microglia in the CNS contribute to neuroinflammation with their inflammatory responses, in which NLRP3 modulation has been reported to play a key role in neurodegenerative diseases. For example, Ji et al. [39] reported that the TLR4 pathway and NLRP3 inflammasome activation are both involved in the M1 polarization, while the NLRP3 inflammasome inactivation by NOSH-NBP switches the microglial phenotype from M1 to M2.

Furthermore, NLRP3 inflammasome deficiency shifts microglial cells towards an M2 phenotype resulting in a reduced amyloid-β deposition in the APP/PS1 model of AD [32]. Our results show that Vit C administration is accompanied by a switch in microglial responses towards an M2 phenotype as documented by CD163upregulation, as well as by anti-inflammatory cytokine increase, in terms of IL-4 and IL-10 up-regulation.

It was demonstratedthat activated microglia might act as a double-edged sword depending on the phenotype. Whereas the M1 phenotype accelerates the pro-inflammatory mediators, increasing neuronal death, the M2phenotype releases anti-inflammatory products and assures neuronal survival and tissue repair [40]. Activation of TLR4 leads to the subsequent activation of NF-κB, which triggers the transcription of pro-inflammatory cytokines, such as IL-1β, IL-6, and TNF-α [41]. Regarding M1 microglia responses, the production of pro-inflammatory cytokines requires the NLRP3 inflammasome [42,43], in which NF-κB is also responsible for NLRP3 inflammation activation [44].

In our PD model, apart from a moderate microglial reaction after in vivo MPTP administration, we detected a marked astrocyte reaction, thus evidencing a prominent role in the pathophysiology of PD. This observation is in line with other previous reports documenting that whereas microglial reactions seem transient on the first to seventh day, the astrocytic reaction persisted from the first until the twenty-first day in MPTP-induced PD mice [45].

In this regard, the NLRP3 inflammasome is expressed in both microglia and astrocytes [46] and contributes to neuronal degeneration [47].

## 5. Conclusions

In conclusion, this study showed the neuroprotective effects of Vit C through the modulation of TLR4/NF-κB/NLRP3/IL-1β in an MPTP murine model of PD. More importantly, we demonstrate for the first time that the use of Vit C reduces neuroinflammation by modulating microglial responses and astrocyte activation, thus suggesting that the inhibition of NLRP3 activation may present promising potential forthe prevention and alleviating of the inflammatory cascade of PD.

## Figures and Tables

**Table 1:** List of primers sequences usedfor PCR amplification.

cDNA Target	Sequence (5′-->3′)	Sequencereferences
TH	FW: TACTTTGTGCGCTTCGAGGT RW: CGTGGCGTATACCTCCTTCC	NM_009377.2
GFAP	FW: CACCTACAGGAAATTGCTGGAGG RW: CCACGATGTTCCTCTTGAGGTG	XM_030245571.2
IBA1	FW: TCTGCCGTCCAAACTTGAAGCC RW: CTCTTCAGCTCTAGGTGGGTCT	XM_006523503.5
TLR4	FW: TGCTTGGCGAATGTTTCTGC RW: TCTGTTCCTTGACCCACTGC	NM_021297.3
TNF-α	FW: GGCAGGTCTACTTTGGAGTCATTGC RW: ACATTCGAGGCTCCAGTGAATTCGG	NM_013693.2
IL-6	FW: CTGGTGACAACCACGGCCTTCCCTA RW: ATGCTTAGGCATAACGCACTAGGTT	DQ_788722.1
iNOS	FW: CACCTTGGAGTTCACCCAGT RW: ACCACTCGTACTTGGGATGC	NM_010927.4
IL-10	FW: TAACTGCACCCACTTCCCAG RW: AGGCTTGGCAACCCAAGTAA	NM_010548.2
IL-4	FW: ATCATCGGCATTTTGAACGAGGTC RW: ACCTTGGAAGCCCTACAGACGA	NM_021283.2
CD40	FW: TTGTTGACAGCGGTCCATCT RW: CTTGCTGGTGCAGTGTTGTC	XM_006499154.4
TGF-β	FW: TGATACGCCTGAGTGGCTGTCT RW: CACAAGAGCAGTGAGCGCTGAA	AJ009862.1
CD163	FW: GGCTAGACGAAGTCATCTGCAC RW: CTTCGTTGGTCAGCCTCAGAGA	XM_006506800.5
GAPDH	FW: ACCACAGTCCATGCCATCAC RW: TCCACCACCCTGTTGCTGTA	BC_085315.1

## Data Availability

The authors declare that all data supporting the findings of this study are available within the paper. All other information is available from the corresponding authors uponrequest.

## Supplementary Materials

The following are available online at https://www.mdpi.com/article/10.3390/biology10111155/s1, Figure S1: The full western blots of Figure 1; Figure S2: The full western blots of Figure 2; Figure S3: The full western blots of Figure 3; Figure S4 (A–E): The full western blots of Figure 6; Figure S5 (A–D): The full western blots of Figure 8; Figure S6 (A–B): The full western blots of Figure 10.
