# Supplementary figures and images for: Inflammatory Response Modulation by Vitamin C in an MPTP Mouse Model of Parkinson’s Disease
Source: Biology (Basel). 2021 Nov 9;10(11):1155. doi: 10.3390/biology10111155 (PMC8614932; doi:10.3390/biology10111155)

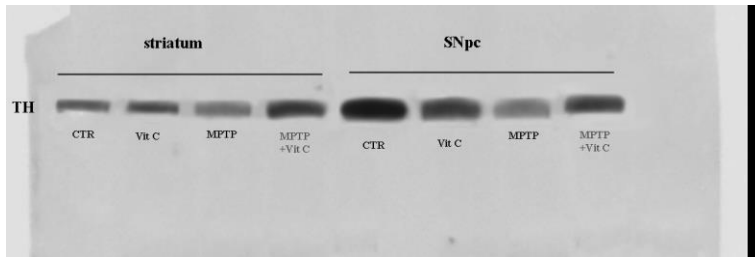

Figure S1

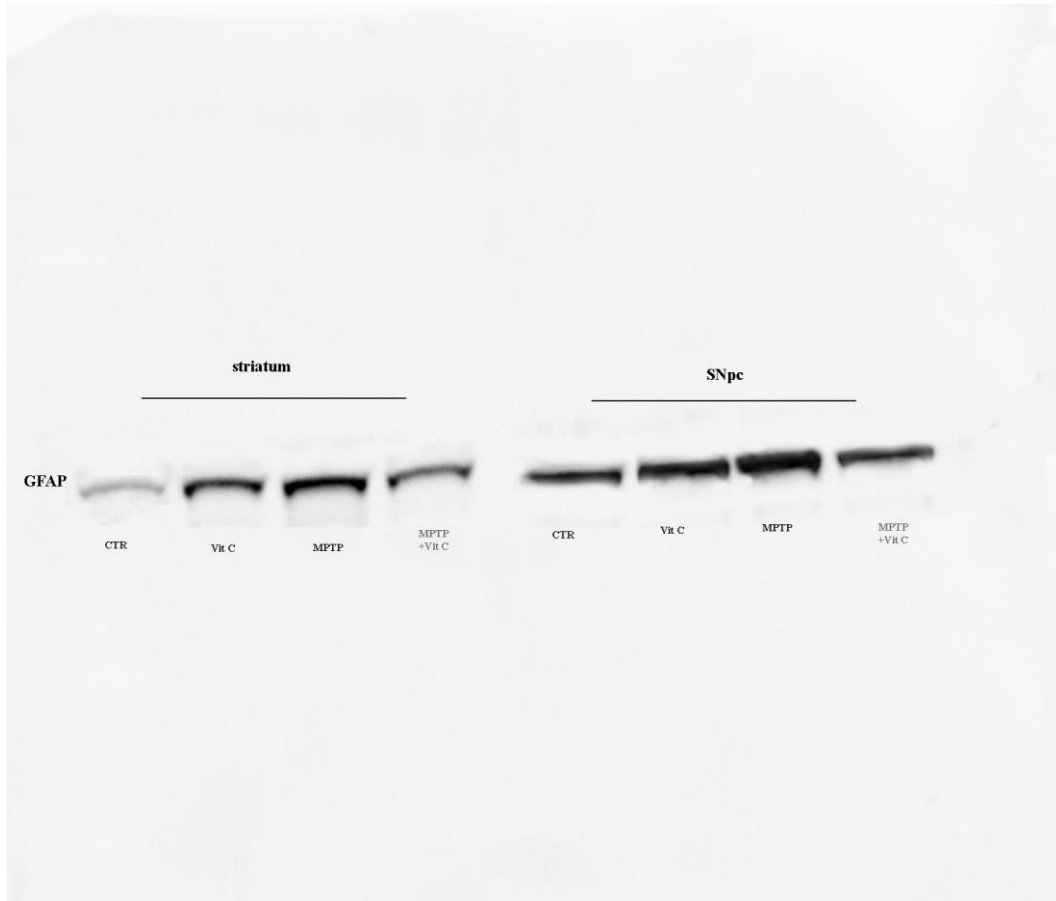

Figure S2

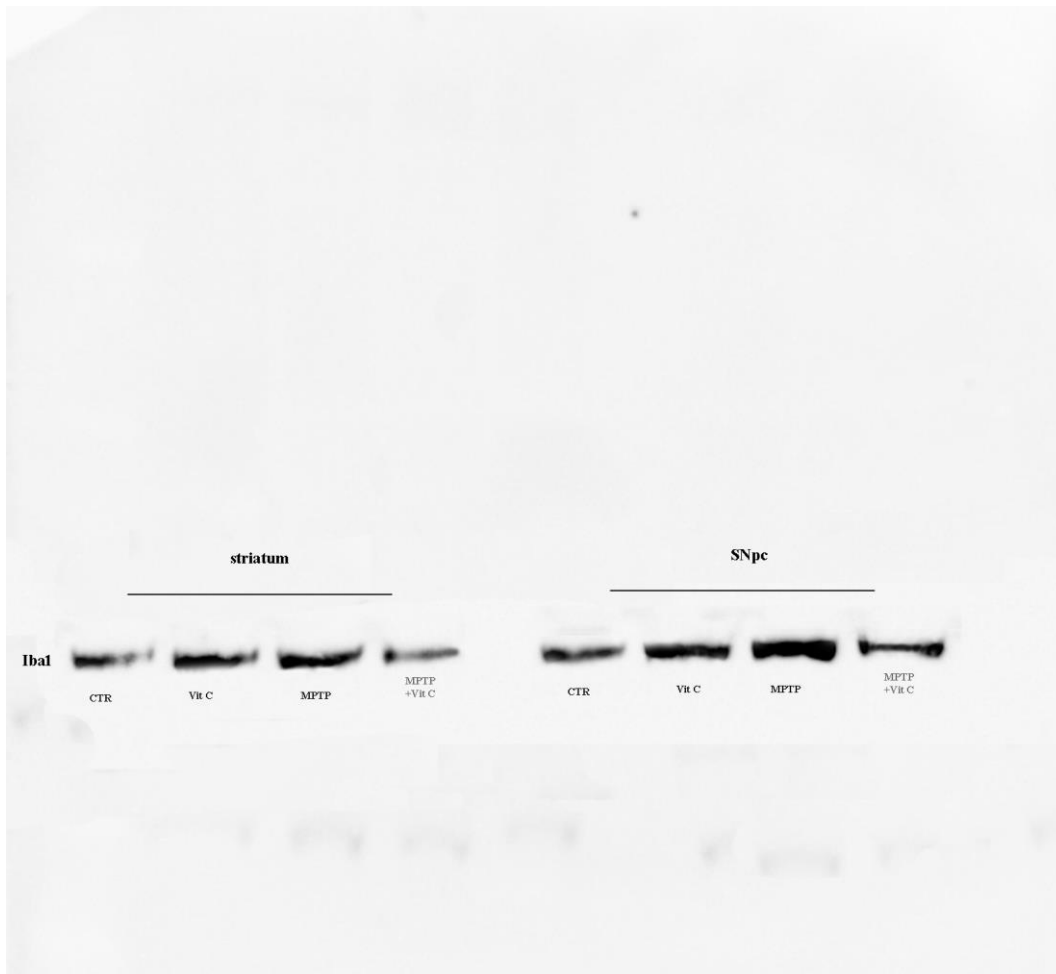

Figure S3

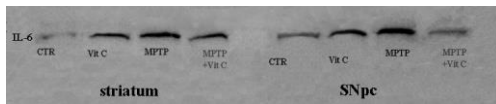

(a)

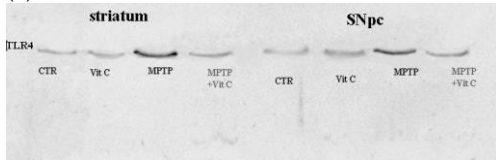

(b)

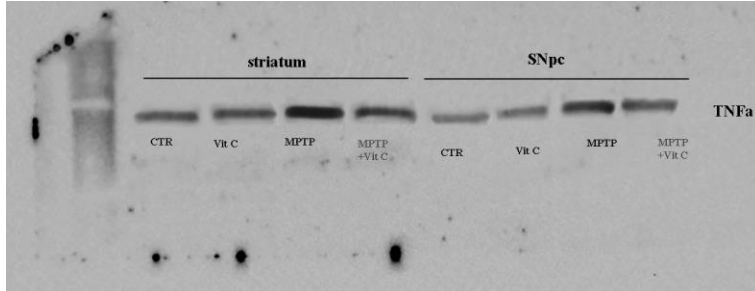

(c)

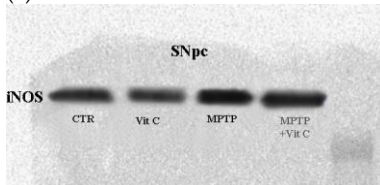

(d) 1

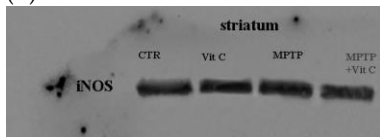

(d) 2

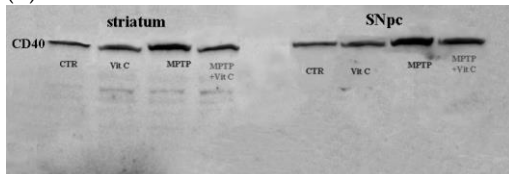

(e)

Figure S4

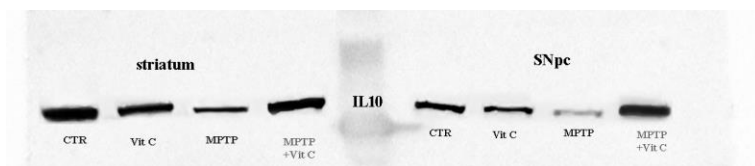

(a)

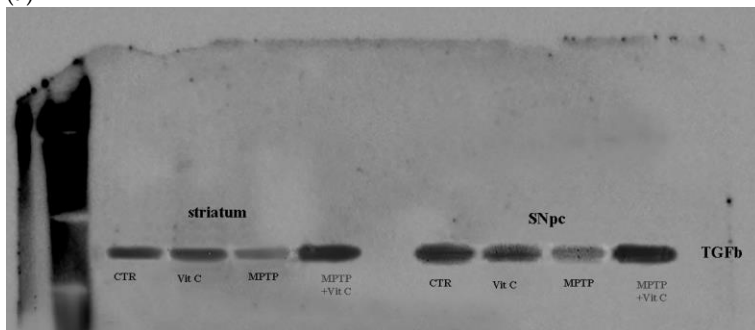

(b)

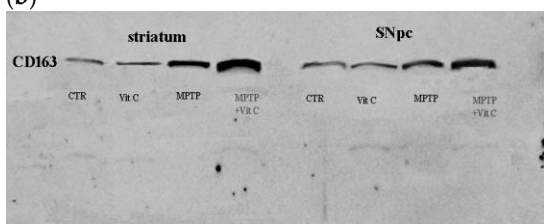

(c)

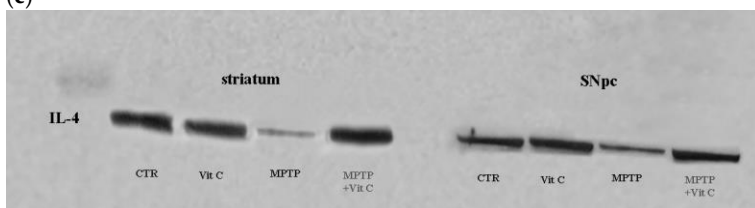

(d)

Figure S5

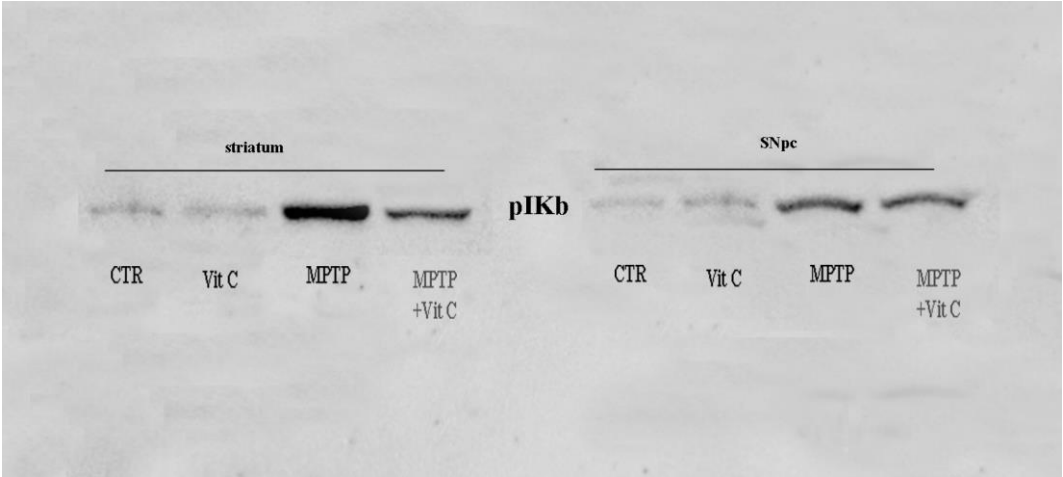

(a)

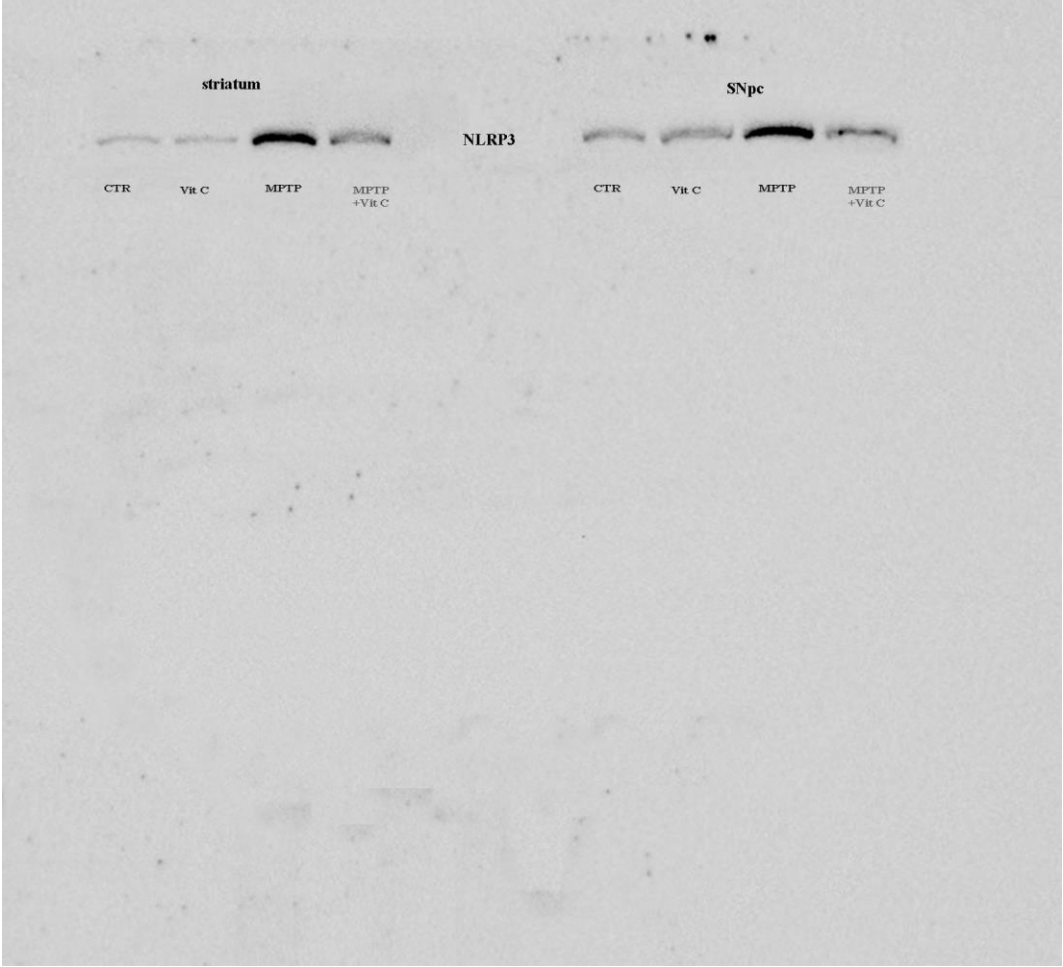

(b)

Figure S6

Supplement: Supplementary file 1 [file biology-10-01155-s001.zip › biology-1435159-supplementary.pdf]

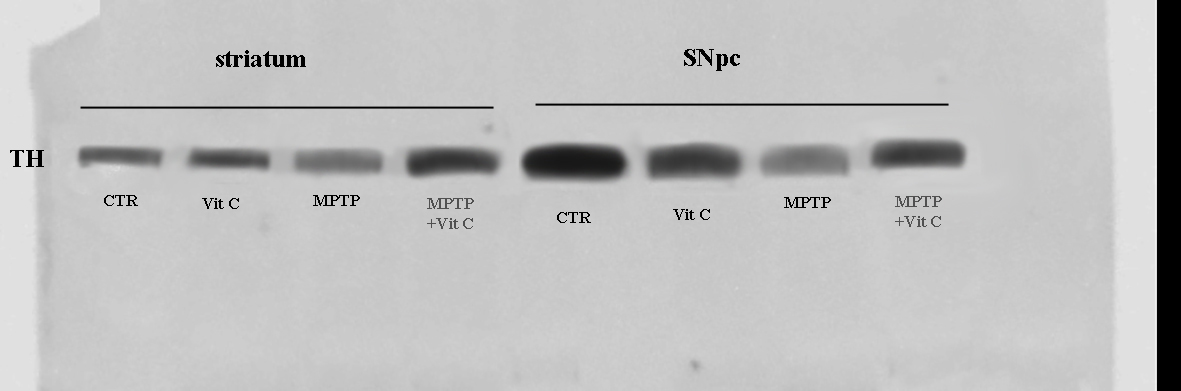

Supplement: Supplementary file 1 [file biology-10-01155-s001.zip › fig S1.jpg]

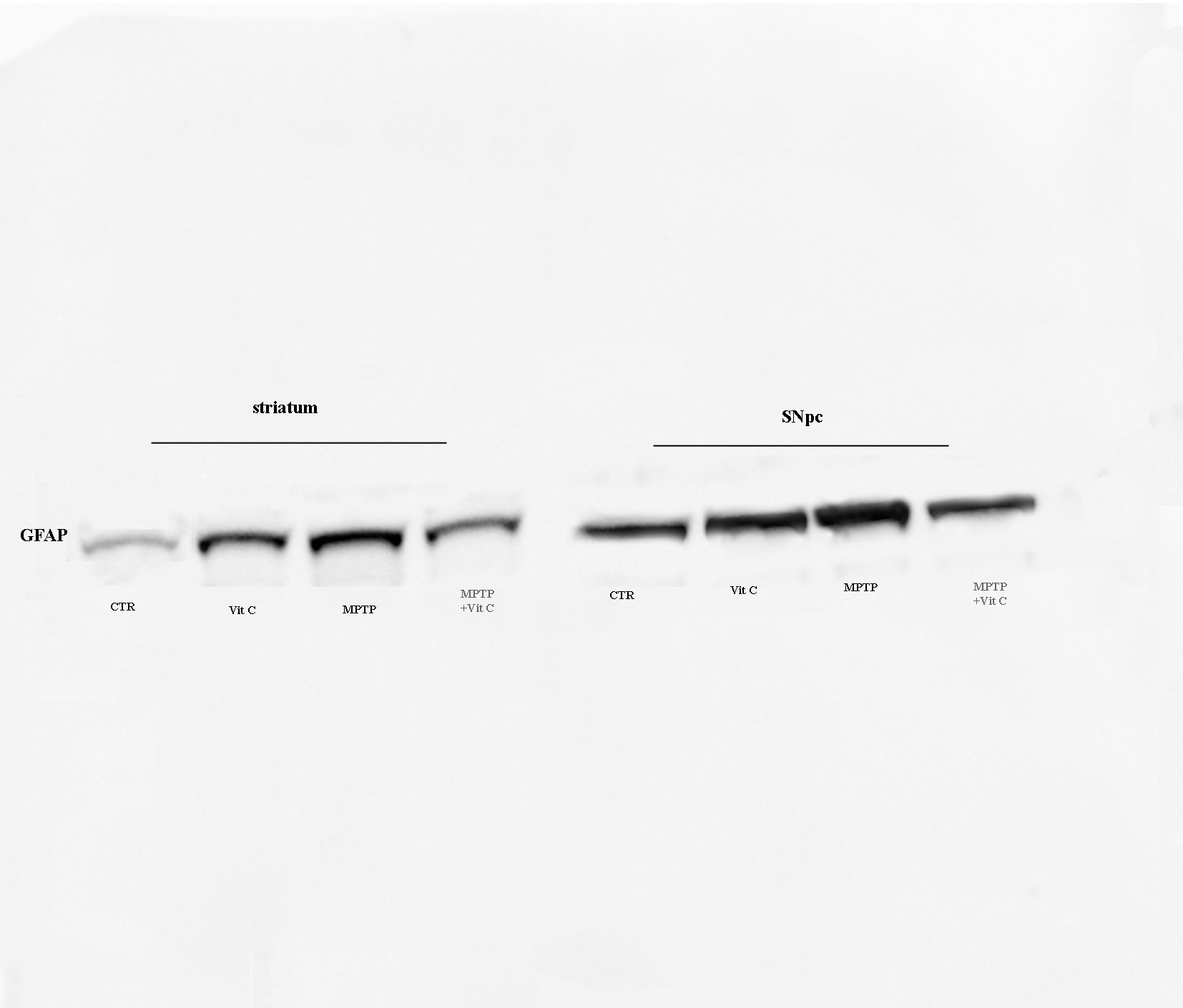

Supplement: Supplementary file 1 [file biology-10-01155-s001.zip › FIG s2.jpg]

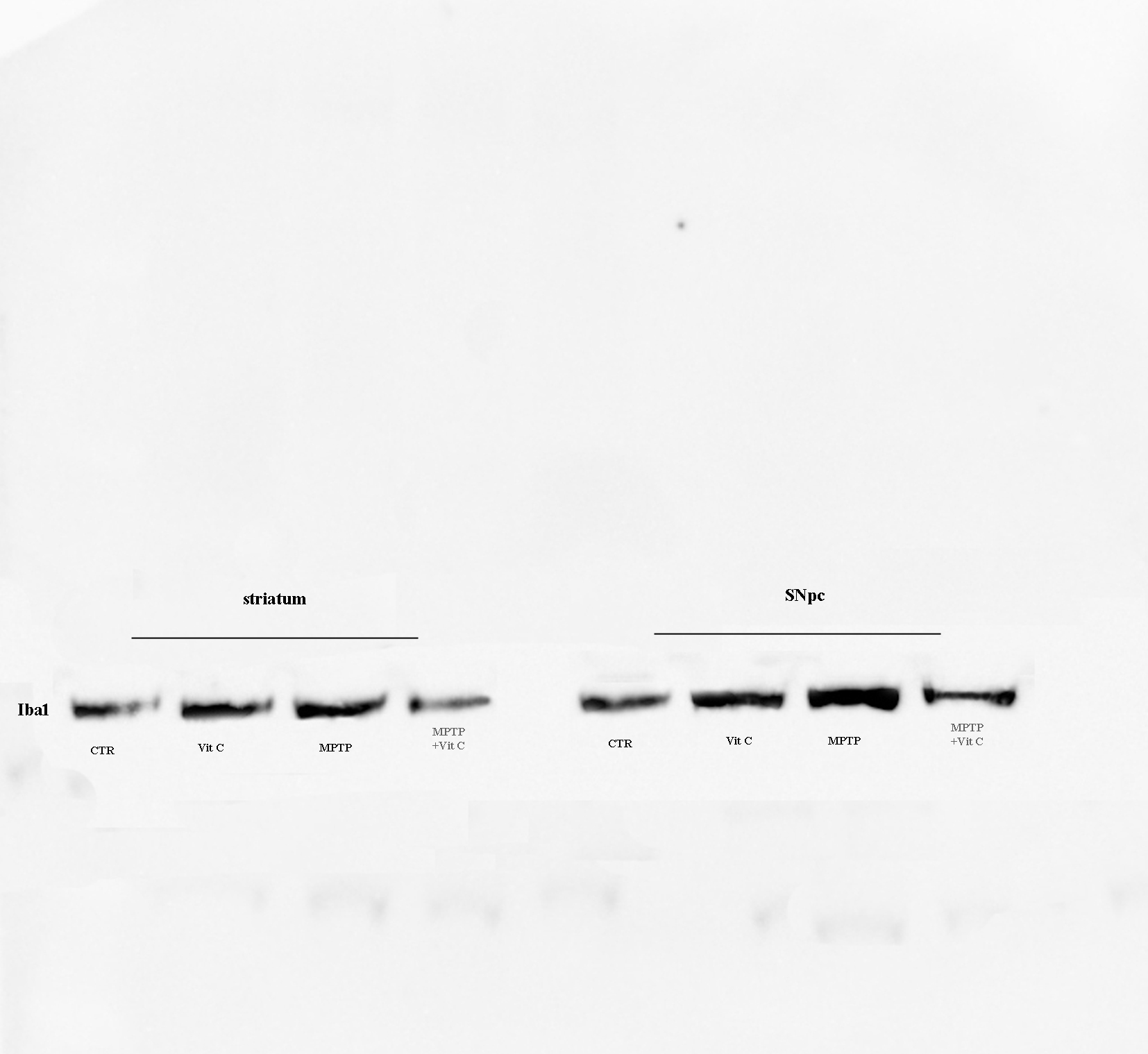

Supplement: Supplementary file 1 [file biology-10-01155-s001.zip › fig s3.jpg]

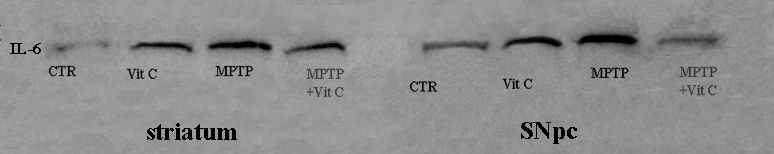

Supplement: Supplementary file 1 [file biology-10-01155-s001.zip › FIG S4A.jpg]

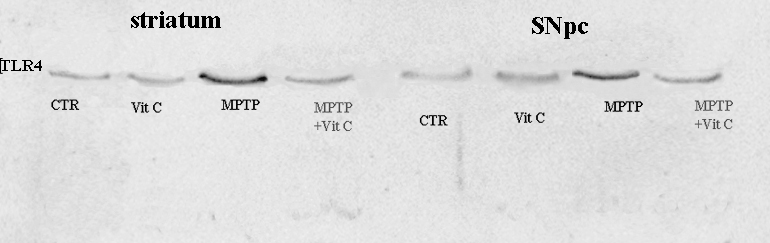

Supplement: Supplementary file 1 [file biology-10-01155-s001.zip › FIG S4B.jpg]

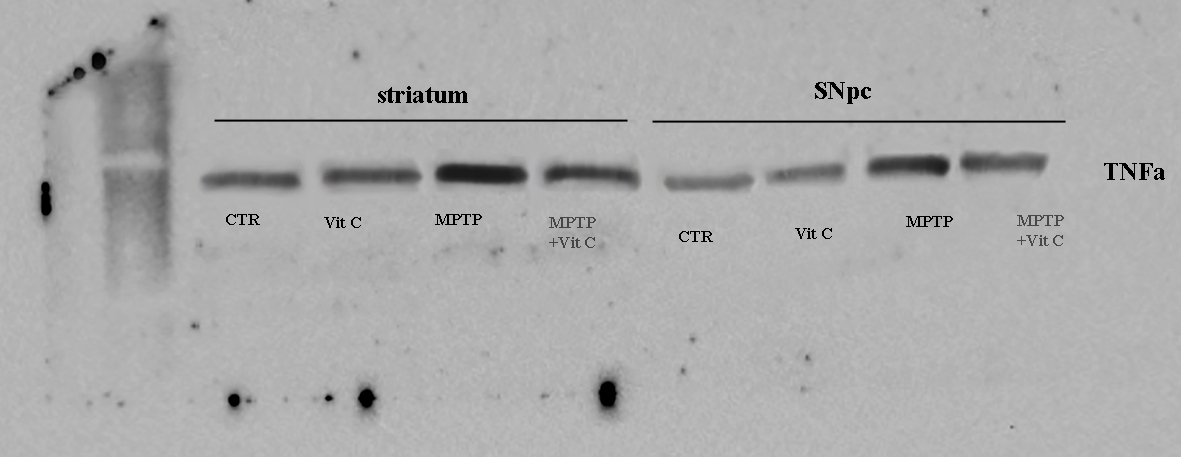

Supplement: Supplementary file 1 [file biology-10-01155-s001.zip › FIG S4C.jpg]

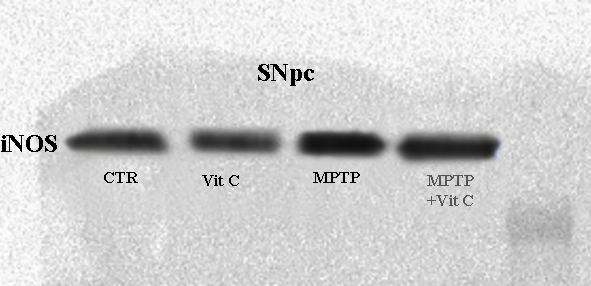

Supplement: Supplementary file 1 [file biology-10-01155-s001.zip › FIG S4D1.jpg]

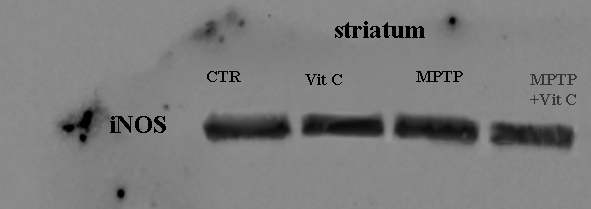

Supplement: Supplementary file 1 [file biology-10-01155-s001.zip › FIG S4D2.jpg]

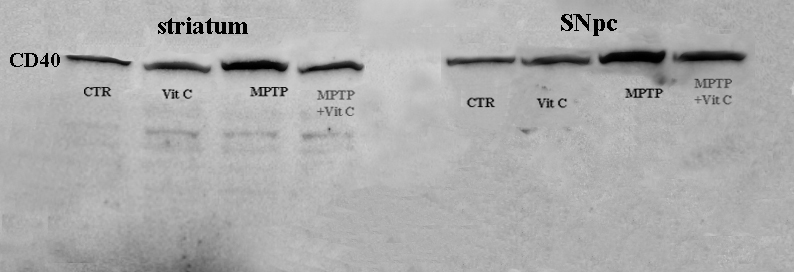

Supplement: Supplementary file 1 [file biology-10-01155-s001.zip › FIG S4E.jpg]

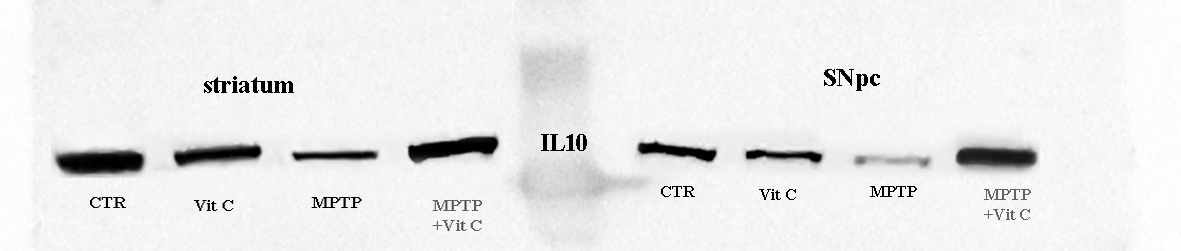

Supplement: Supplementary file 1 [file biology-10-01155-s001.zip › FIG S5A.jpg]

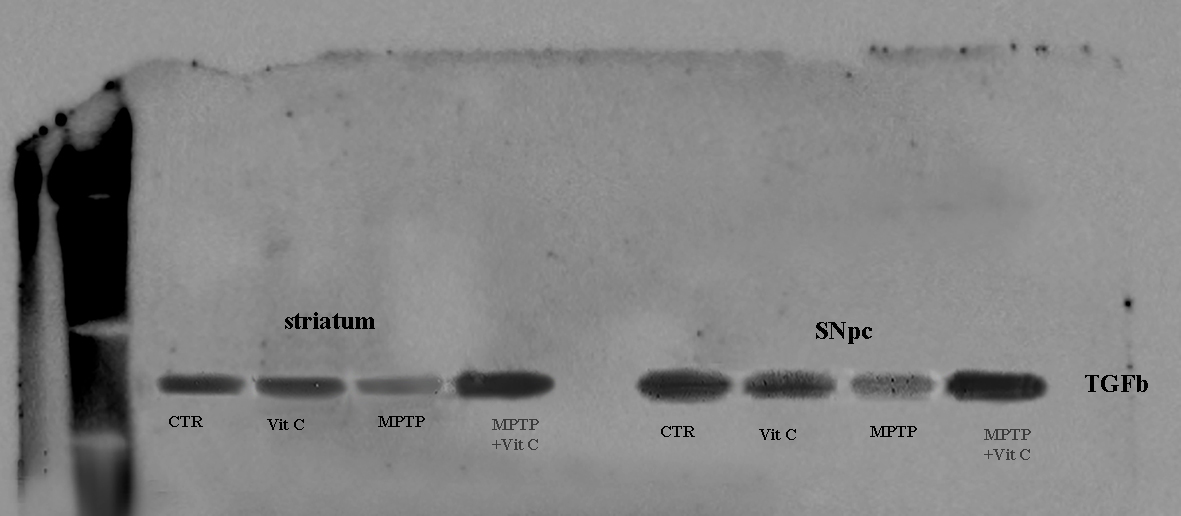

Supplement: Supplementary file 1 [file biology-10-01155-s001.zip › FIG S5B.jpg]

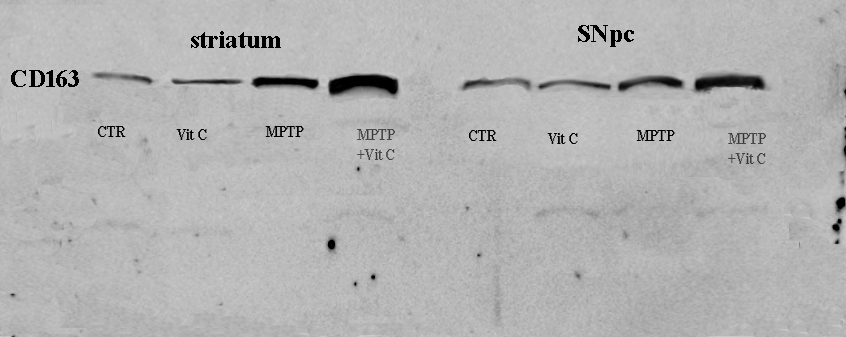

Supplement: Supplementary file 1 [file biology-10-01155-s001.zip › FIG S5C.jpg]

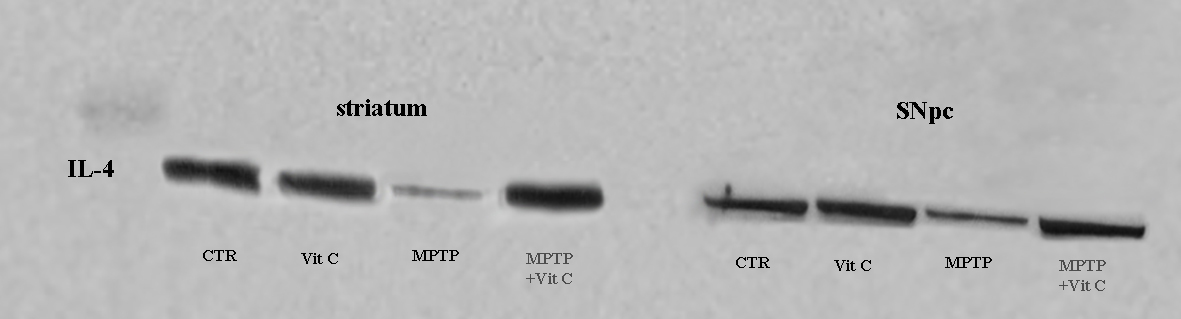

Supplement: Supplementary file 1 [file biology-10-01155-s001.zip › FIG S5D.jpg]

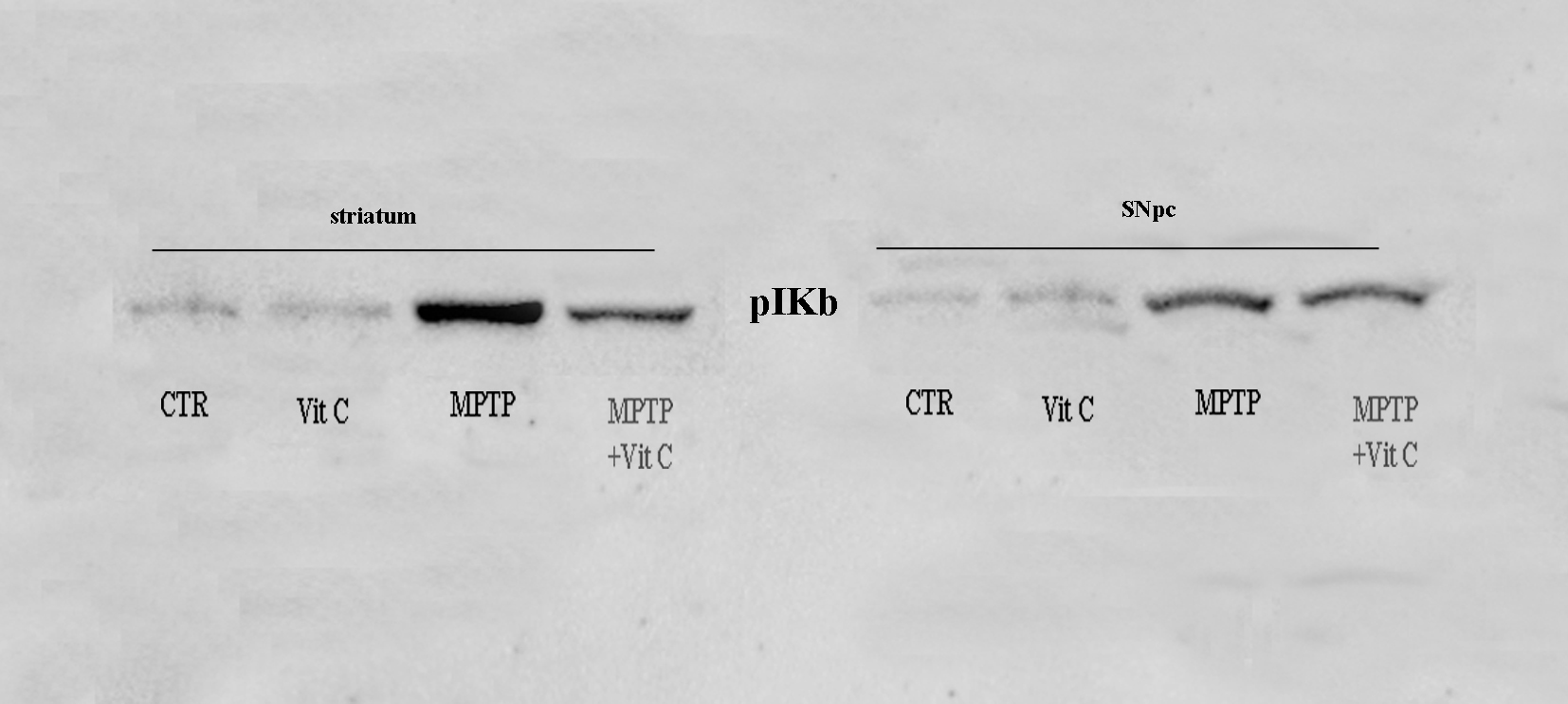

Supplement: Supplementary file 1 [file biology-10-01155-s001.zip › FIG S6A.jpg]

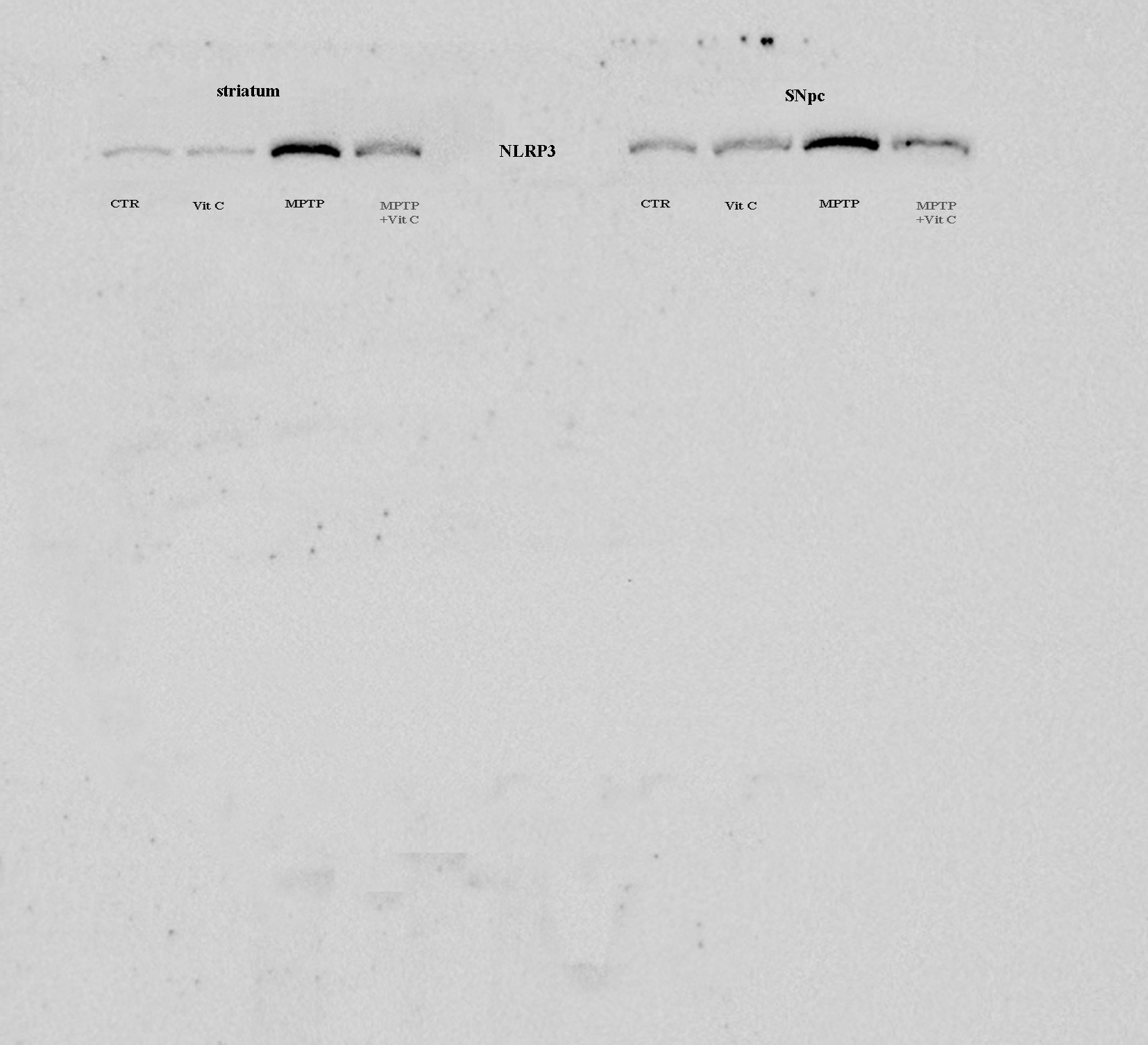

Supplement: Supplementary file 1 [file biology-10-01155-s001.zip › FIG S6B.jpg]
